# Investigation of Cytotoxic Activity in Four *Stachys* Species from Iran

**Published:** 2012

**Authors:** Mahnaz Khanavi, Azadeh Manayi, Mahnaz Lotfi, Rofeyde Abbasi, Maryam Majdzadeh, Seyed Nasser Ostad

**Affiliations:** a *Department of Pharmacognosy, Faculty of Pharmacy and Traditional Iranian Madicines and Pharmacy Research Center*^*.*^*, Tehran University of Medical Sciences, Tehran, Iran.*; b *Pharmaceutical Sciences Branch, Islamic Azad University, Tehran, Iran.*; c *Department of Toxicology and Pharmacology, Faculty of Pharmacy and Pharmaceutical Sciences Research Center, Tehran University of Medical Sciences, Tehran, Iran.*

**Keywords:** Cytotoxic activity, Stachys laxa Boiss. and Buhse, Stachys turcomanica Trautv, Stachys subaphylla Rech. F, Stachys trinervis Aitch. and Hemsl., MTT assay

## Abstract

The aerial parts of *Stachys laxa *Boiss. and Buhse. from Siah-bishe in Mazandaran province, *Stachys trinervis* Aitch. and Hemsl. from Karaj in Alborz province, *Stachys subaphylla *Rech. F. and *Stachys turcomanica* Trautv. from Golestan province have been collected in May 2008. Total extracts were obtained through MeOH/H_2_O (80/20) and then partitioned between CHCl_3_, EtOAc and MeOH. These fractions and total extracts have been investigated for *in-vitro* cytotoxic activity against the colon carcinoma (HT-29), colorectal adenocarcinoma (Caco-2), breast ductal carcinoma (T47D) and Swiss mouse embryo fibroblast (NIH 3T3) cell lines using MTT assay (3-(4,5-di methyl thiazol-2-yl)-2,5-di phenyltetrazolium bromide). At each cell line, doses of 3.125, 6.25, 12.5, 25, 100, 200, 400 and 800 µg/mL in 1% (v/v) DMSO of all samples were tested. Ethyl acetate and chloroform fractions of* Stachys laxa* against proliferation of T47D and HT-29 cell lines and chloroform fraction of* Stachys subaphylla* and *Stachys subaphylla *ethyl acetate fraction toward T47D cell line exhibited highest cytotoxic activity (IC_50_ < 50 µg/mL). Ethyl acetate and chloroform fractions of* Stachys turcomanica *against HT-29 cell line, except methanol fraction of *Stachys subaphylla, *the other extrcts on T47D cell line, represented moderate cytotoxic activity (IC_50_ < 70 µg/mL). All fractions of *S. trinervis* demonstrated no effective cytotoxic activity. IC_50_ values confirmed that the growth and proliferation of HT-29 and T47D cells were most affected by chloroform and ethyl acetate fractions of* Stachys laxa *and* Stachys turcomanica* due to their nonpolar compounds.

## Introduction

The genus *Stachys* belongs to the plant family of Lamiaceae. The most species of this genus has been previously analyzed in numerous studies concerning their chemical composition, pharmacological properties and therapeutic uses. This family is well represented in the flora of Iran, at least with 200-300 species in the world (1) and 34 species in Iran (2). Phytochemical investigation of some *Stachys* species has demonstrated phenolic acids, tannins (3, 4), flavonoids (5) and phenyl ethanoid glycosides (6, 7). There are some reports about pharmacological activities of this genus including anticancer (8, 9), antibacterial (10- 13), antioxidant effects (14- 16), anti-inflammatory (17-22), anti-nephritic (23) and anti-anxiety (24). Some *Stachys* species are used in folk medicine for healing wounds, disinfectant, treating abdominal pains, asthma, rheumatic and inflammatory disorders, anti-spasmodic and anti-fever (20, 25). Essential oils of *Stachys cretica* ssp. *lesbiaca* Rech. fil. and *S. cretica* ssp. *trapezuntica* Rech. fil. inhibit the growth of HL-60 and Ishikawa human tumor cell lines; the main component in both of them was germacrene D (26). There was an investigation about the essential oil of *S. turcomanica*; the major constituents were identified as germacrene D (17.4 %), 7-epi-á-selinene (10.5 %), *β*-elemene (9.2 %) and *β*-pinene (8.6 %) (27). Major components of *S. trinervis* oil were identified as *α*-pinene (42.68 %), *δ*-2-carene (31.90 %), 1,8-cineole (7.03 %), limonene (4.39 %) and (Z)-*β*-ocimene (4.21 %). *S. subaphylla* oil major constituents were *δ*-2-carene (23.93 %), *α*-pinene (19.29 %), sabinene (19.11 %), *δ*-3-carene (9.22 %) and (Z)-*β*- ocimene (5.90 %) (28). Thirty three constituents from essential oil of *S. laxa* have been identified and the major constituents were germacrene D (40.1%), *β*-caryophylene (16.7%), *β*-phellandrene (5.5%), caryophyllene oxide (4.6%), linalool (3.2%) and *α*-cadinol (2.6%) (29). The cytotoxicity of some *Stachys* species against A431, HeLa and MCF-7 were examined. *S. recta* and *S. palustris* stem extract inhibit the growth of HeLa cells. An* S. rectum *was significantly active toward the breast MCF7 cell line (30). More than half of drugs in cancer therapy were obtained from natural products or related to them (31). Hence in this study, cytotoxic activity of *S. laxa* Boiss. and Buhse., *S. turcomanica* Trautv, *S. subaphylla* Rech. F., *S. trinervis* Aitch. and Hemsl. have been investigated toward four cell lines by MTT assay.

## Experimental

*Plant material*

The aerial parts of *S. laxa *Boiss. and Buhse., from Siah-bishe in Mazandaran province, *S. trinervis* Aitch. and Hemsl from Karaj in Alborz province, and *S. subaphylla *Rech. F. and *S. turcomanica* Trautv. from Golestan province were collected in May 2008. The plants have been identified and deposited at the Herbarium of Faculty of Pharmacy, Tehran University of Medical Sciences, Tehran, Iran.

*Extraction*

Freshly collected aerial parts of four species of *Stachys* were cleaned and shade dried. These parts were coarse powdered in a hand mill and stored at room temperature. Two hundred grams of powdered plants were extracted through perculation method with 80% aq. MeOH three times at room temperature. The extract was evaporated using rotary evaporator and consequently partitioned between CHCl_3_, EtOAc and MeOH. Each fraction evaporated with rotary evaporator and has been stored at refrigerator for the investigation of cytotoxic activity.

**Table 1:** Cytotoxic activity of total extract and fractions of four species of *Stachys*

**Samp**le	**Cell Lines** ^a^ ** (MTT assay)**			
	**HT-29**	**Caco-2**	**T47D**	**NIH/ 3T3**
*Stachys laxa*				
Total extract	421.97 ± 8.71	> 1000	239.78 ± 16.92	508.77 ± 45.56
Methanol fr.	265.83 ± 46.52	> 1000	254.1 ± 7.45	405.7 ± 74.18
Ethyl acetate fr.	134.004 ± 1.764	116.53 ± 18.23	18.079 ± 2.248	31.452 ± 1.554
Chloroform fr.	27.007 ± 2.096	101.65 ± 12.4	21.106 ± 2.491	41.294 ± 8.391
*Stachys subaphylla*				
Total extract	> 1000	> 1000	60.15 ± 3.72	771.26 ± 164.67
Methanol fr.	> 1000	> 1000	771.26 ± 197.38	> 1000
Ethyl acetate fr.	-	116.52 ± 2.78	51.05 ± 8.89	-
Chloroform fr.	234.86 ± 11.28	183.85 ± 8.87	43.411 ± 9.99	74.27 ± 2.34
*Stachys trinervis*				
Total extract	> 1000	> 1000	358.1 ± 14.14	> 1000
Methanol fr.	> 1000	> 1000	630.96 ± 29.99	649.23 ± 17.91
Ethyl acetate fr.	241.66 ± 14.71	338.22 ± 1.02	128.35 ± 6.65	110.05 ± 5.56
Chloroform fr.	> 1000	> 1000	383 ± 4.01	674.84 ± 67.37
*Stachys turcomanica*				
Total extract	219.58 ± 14.21	> 1000	103.67 ± 12.43	308.7 ± 1.34
Methanol fr.	693.57 ± 56.91	> 1000	708.60 ± 25.8	802.58 ± 26.84
Ethyl acetate fr.	66.10 ± 5.43	87.08 ± 8.9	30.14 ± 1.78	50.45 ± 3.45
Chloroform fr.	66.84 ± 7.92	187.89 ± 11.72	51.38 ± 9.49	58.22 ± 4.06
*Methotrexate*	0.23 ± 0.02	0.32 ± 0.04	0.16 ± 0.09	0.24 ± 0.013

^a^Results are expressed as IC_50_ values (μg/mL), Key to cell Lines employed: HT-29 and Caco-2 (colon Adenocarcinoma), T47D (breast carcinoma), NIH 3T3 (Swiss embryo fibroblast.

*Cytotoxicity assay*

The colon carcinoma (HT-29), colorectal adenocarcinoma (Caco-2) and ductal carcinoma (T47D) cell lines were mentioned as exponentially growing cultures in RPMI 1640 cell culture medium (PAA, Germany), supplemented with 10% fetal bovine serum (FBS: Gibco, USA), for HT-29 cells and 15% FBS for Caco-2 and T47D cells. The Swiss mouse embryo fibroblast (NIH 3T3) cell line was kept in Dulbecco’s modified Eagle’s medium (DMEM; PAA, Germany) supplemented with 10% FBS. 100 IU/mL penicillin and 100 µg/mL streptomycin (Roche, Germany) were added to the media. All the cell lines were cultured at 37°C in air /carbon dioxide (95:5) atmosphere.

Cytotoxic activity was measured using modified MTT assay (31). 1×10^4 ^cells/well were plated in 96-well plates (Nunc, Denmark) and incubated for 24 h before the addition of drugs. After 96 h of incubation in Caco_2 _cells and 48 h of incubation in HT-29, NIH/3T3 and T47D cells, 20 µL of MTT (Merck, Germany) reagent (5 mg/mL) in phosphate buffered saline (PBS) was added to each well. The plates were incubated at 37°C for 4 h. The medium was discharged and the formazan blue, which had been formed in the cells, were dissolved with 100 µL dimethyl sulphoxide (DMSO). After the incubation at 37°C for 10 min, absorbance at 570 nm at the dissolved solutions was detected using a micro plate reader (Anthos, Austria). The cell viability in MTT assay was calculated as the percentage of control value. Methotrexate was used as the positive control. Cytotoxicity was expressed as the concentration of extract inhibiting cell growth with 50% (IC_50_ ± SD). All tests and analysis were run in triplicate.

*Statistical analysis*

IC_50 _(the median growth inhibitory concentration) values were calculated from the IC_50 _of dose-response curve in the sigma plot 11 software. Data representative of three independent experiments with similar results were presented as mean ± SD.

## Results

The effects of these plant extracts on the proliferative response of the HT-29, Caco-2 and T47D cell lines have been analyzed by treating the cells with different concentrations of the extracts and significant decrease in cell lines proliferation were observed. IC_50_ ± SD are reported in Table 1. The chloroform and ethyl acetate fractions of *S. laxa *Boiss. showed high cytotoxicity on T47D, HT-29 (IC_50_ < 50 µg/mL). The cytotoxicity of ethyl acetate fraction of *S. turcomanica* Trautv. and chloroform fraction of *S. subaphylla *were better than the other fractions on T47D cell line (IC_50_ < 50 µg/mL). Total extract and fractions of* S. trinervis* did not affect the cell lines*.*

## Discussion

Among all the samples, nonpolar (chloroform and ethyl acetate fractions) fractions of *S. laxa* exhibited greatest cytotoxicities on T47D and HT-29 cell lines compared with polar fraction and total extract. According to the data, the cytotoxic activity of chloroform and ethyl acetate fractions on HT-29 and T47D cell lines were much stronger than that of Caco-2. It indicated that chloroform and ethyl acetate fractions of *S. laxa* had potential cytotoxic selectivity on T47D cell line. There was a report about the antioxidation and total phenol content of some *Stachys* spp. The research implied that total phenol content and FRAP value of methanolic extract are in this order: *S. laxa* > *S. turcomanica* > *S. subaphylla* > *S. trinervis *(33). Except *S. subaphylla* total extract against T47D cell line, the other methanolic extracts have indicated the same order of cytotoxic activity on T47D and HT-29. Higher cytotoxic activity of nonpolar fraction of *S. laxa* and *S.turcomanica *may be due to the high content of germacrene D in their essential oil, same as the *Stachys cretica* ssp. (34, 35), but main components of *S. subaphylla* and *S. trinervis* essential oils were identified as monoterpene hydrocarbons. In comparison with another fraction, methanolic and total fractions of all samples demonstrated slightly cytotoxic effect on cell line tested. The real IC_50 _values of fractions of four species Stachys may be considerably lower than the positive control (Methotrexate) since its pharmacological active compounds are not pure and further researches are needed for defining potential component as cytotoxic natural medicines.
